# Secure optical communication using a quantum alarm

**DOI:** 10.1038/s41377-020-00409-1

**Published:** 2020-10-02

**Authors:** Yupeng Gong, Rupesh Kumar, Adrian Wonfor, Shengjun Ren, Richard V. Penty, Ian H. White

**Affiliations:** 1 Centre for Advanced Photonics and Electronics, University of Cambridge, 9 JJ Thomson Ave, Cambridge, CB3 0FA UK; 2 Quantum Communications Hub, Information Centre, Department of Physics, University of York, York, YO10 5DD UK; 3 University of Bath, Claverton Down, Bath, BA2 7AY UK

**Keywords:** Fibre optics and optical communications, Quantum optics

## Abstract

Optical fibre networks are advancing rapidly to meet growing traffic demands. Security issues, including attack management, have become increasingly important for optical communication networks because of the vulnerabilities associated with tapping light from optical fibre links. Physical layer security often requires restricting access to channels and periodic inspections of link performance. In this paper, we report how quantum communication techniques can be utilized to detect a physical layer attack. We present an efficient method for monitoring the physical layer security of a high-data-rate classical optical communication network using a modulated continuous-variable quantum signal. We describe the theoretical and experimental underpinnings of this monitoring system and the monitoring accuracy for different monitored parameters. We analyse its performance for both unamplified and amplified optical links. The technique represents a novel approach for applying quantum signal processing to practical optical communication networks and compares well with classical monitoring methods. We conclude by discussing the challenges facing its practical application, its differences with respect to existing quantum key distribution methods, and its usage in future secure optical transport network planning.

## Optical communication security: Sound the quantum alarm

A quantum alarm system can detect eavesdropping on optical communication links faster than classical methods. The system was developed by Yupeng Gong and colleagues at the University of Cambridge, UK, and involves transmitting two modes that are indistinguishable to an eavesdropper over the same channel and at the same wavelength. One mode sends a classical data signal and the other sends the security-monitoring quantum modulated signal. The alarm system is low-cost and can easily be used with high data-rate communication links that are hundreds of kilometers long. It can also be used with other encryption methods. The quantum alarm overcomes issues in other attack detection techniques, including quantum key distribution, which is challenging to implement in high data-rate optical communication networks.

## Introduction

The evolution of current optical communication systems towards highly diverse, flexible networks with broad coverage for mission-critical applications has made channel security a critical issue. As described in ref. ^1^, we may define two types of security in optical communication networks: physical layer security and semantic security. High semantic security ensures that an adversary is not able to compute any communications information from a ciphertext, while physical layer security protects channels by ensuring data privacy.

### Current classical attack detection methods

To date, several fibre surveillance, in-service monitoring or active fibre monitoring methods have been devised^2–5^ to protect channels from physical layer attacks^6–8^. There are generally two categories of attack detection techniques, based on their working principles^7,9^: (1) methods that rely on additional statistical analysis of the communications signals (e.g., mean optical power monitoring, bit error rate (BER) measurement and optical spectrum analysis (OSA)) and (2) methods that rely on sending a special signal devoted to investigative purposes (e.g., optical time-domain reflectometry (OTDR) and pilot tones). The parameters that are monitored to ensure security indicate the degree to which security is violated.

Methods of the first kind are often too slow to detect an attack that lasts for only a few seconds^6^. In addition, it is possible to maintain the link power while splitting off part of the information using a correlated jamming attack^9,10^. For a method of the second kind, if the act of attack causes significant degradation of the probe signal, then the tapped channel will also be affected, and vice versa^6,11^. In addition, although OTDR^12^ can locate a fault in a channel, its sensitivity (0.01 dB/km^4^ or 0.5 dB/dB loss^3^) is usually too poor to detect a sophisticated eavesdropper, who will usually cause a loss change of less than 0.1 dB^1^ at a long distance. Regarding jamming attacks, none of the above methods is sensitive to channel noise, and they cannot detect a jamming attack, unless the noise causes significant degradation of the signal, resulting in many corrupted bits, or the noise significantly affects the pilot tone/probe signal.

### Quantum techniques for secure communication

On the other hand, quantum techniques, particularly those focused on quantum key distribution (QKD)^13,14^ and quantum secure direct communication (QSDC)^15–17^, seek to ensure security by using information-theoretical secure techniques rather than by relying on computational complexity. A fundamental problem with classical monitoring, as analysed in^18,19^ and experimentally realized in^20^, is that the classical nature of current optical communication signals allows an attacker to eavesdrop and then resend an identical replica without detection by legitimate users. This kind of attack is known as a man-in-the-middle attack or an intercept-resend attack.

In contrast, quantum communication techniques, which employ the no-cloning theorem^21^, are able to eliminate the threat of this kind of attack. For instance, both QKD, which is capable of distilling secret key material with an arbitrarily small upper bound on the amount of information that is accessible to an eavesdropper, and QSDC, which conveys secure information or deterministic key information directly^22^ based on Wyner’s wiretap theory^23^, consist of an error-check or error estimation step, in which legitimate users are able to check for the presence of any eavesdropper on a quantum communication channel using part of the quantum signals received before the distillation of a secure key or the communication of a secure message.

### Quantum techniques for physical layer security

There are also applications that use quantum techniques to protect physical layer security^24^. proposes a quantum method for protecting line-of-sight channel security. Alternatively, in ref. ^18^, the system monitors the security of the physical layer via a separate reference channel by performing an entanglement test on the received photons, something that is difficult to implement in practice. In^25^, quantum data locking is used to transmit messages at a higher rate with compromised security^26^. proposes a theoretical method of confidential communication using continuous-variable quantum states^27^, in which part of the sent quantum states are used to monitor the security of an ideal channel. Recently, efforts have also been made to use quantum techniques to protect high-data-rate classical communication, e.g., using quantum low probability of intercept^28^ and a spectral approach^29^.

In this paper, therefore, we report a novel technique that we call a quantum alarm (QA), which focuses on monitoring the physical layer security of optical fibre links using quantum techniques. Unlike QKD systems, which are challenging to implement in high-data-rate classical optical communication networks, a QA system can be integrated directly into a high-speed classical communication system, even one that incorporates optical amplifiers. It provides efficient, real-time, long-distance, and low-cost security monitoring. In this work, the QA concept is implemented using a technique relying on continuous variable-(CV) based quantum communications^30^, as this allows equipment similar to that applied in classical coherent communications systems to be used and hence allows the envisaged system to be low in cost.

## Results

### Monitoring principle and protocol

In a method similar to that used in pilot tone systems, the link security is checked by sending special signals, which, in this case, comprise CV quantum states, i.e., weak coherent states modulated at the quantum level. They are sensitive to any unauthorized measurement in the channel, which will be detected, as this introduces extra noise.

**Fig. 1:**
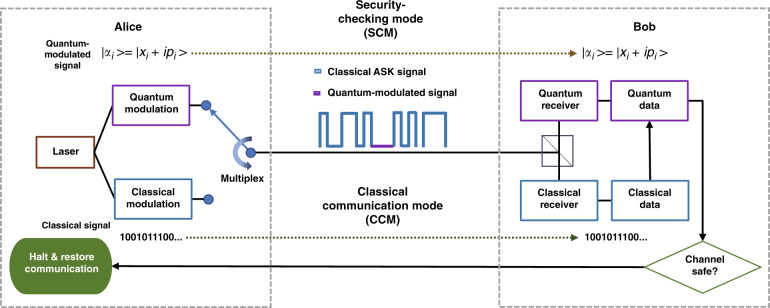
Block diagram of an example quantum alarm system. The blue signal represents a classical signal using an amplitude shift keying (ASK) modulation scheme. The violet signal represents a displaced quantum-modulated signal whose length is less than that of the maximum number of sequential zeros permitted in the classical modulation scheme. The quantum transmitter consists of an amplitude and phase modulator, while the quantum receiver is a low-bandwidth homodyne/heterodyne detector. A splitter is used at the receiver such that both the quantum and classical receivers measure the incoming signal. The system switches between transmitting the classical signal and the quantum signal. The quantum signal is used to check the link security

Hence, as illustrated in Fig. 1, our proposed system has two modes: (i) when sending a quantum-modulated signal, the system is in the security checking mode (SCM), and (ii) it is in the classical communication mode (CCM) when sending classical data signals.

To make these two modes indistinguishable by an eavesdropper without attacking the quantum signal, one may transmit both modes simultaneously, as described in ref. ^31^ and experimentally realized in ref. ^32^, in which QKD and classical coherently modulated signals were transmitted simultaneously using a displaced quantum signal so that there would be no question of distinguishability. However, the small bandwidth and the measurement range of a typical quantum detector limit the practical application of such a system. Hence, in this work, the transmitter switches randomly between the SCM and CCM using optical time-division multiplexing (OTDM). Moreover, we also send the signals over the same channel and at the same wavelength. Given its very low intensity, the quantum-modulated signal should be amplitude displaced in the phase space to increase its intensity to the classical level of zeros in classical communication. As a result, to an eavesdropper, the quantum signal will appear as a short burst of zeros. To further increase the indistinguishability, some additional short bursts of zeros could be introduced during the CCM. Alternatively, Alice could insert quantum signals by replacing all classical zeros such that an eavesdropper cannot identify the SCM by looking for zeros. One could further increase the intensity of the quantum-modulated signal at the price of additional detection complexity. A detailed analysis on this topic can be found in the Supplementary Information.

The receiver uses either homodyne or heterodyne detection to measure the either in phase or quadrature components of the coherent states (X or P) individually or both. A strong local oscillator (LO) pulse (more than 10^8^ photons per pulse), which can be transmitted with the signal or generated locally at the receiver^33^, is employed to detect the information encoded in the quantum modulations.

The information stored in the SCM, along with its position, will be sent via the CCM after a short period of time. The classical receiver decodes the information and passes it to the quantum receiver, which then retains only the measurement results for the quantum signal. This procedure is designed to avoid sending additional header information that reveals the slot information, which may introduce security vulnerabilities. Since no restrictions are placed on the classical detection system in the QA system, the classical channel can have a much higher data rate.

The security is continuously checked by comparing the quadrature values encoded in the quantum state received by Bob and sent by Alice. An attack is found to have taken place when the excess quantum noise *ξ* and the real-time channel transmittance *T* estimated from the quantum states exceeds a given threshold set by the user. Once the link is regarded as unsafe after the SCM, the succeeding CCM is halted, and communication is restored using an alternative secure link in the network.

As mentioned in the introduction, this method of security checking is also employed in QKD and QSDC. However, data reconciliation^34^ and privacy amplification^35^ for key generation are not required. In addition, in QKD, only part of Alice’s quadratures are revealed to Bob for parameter estimation, while in QA monitoring, all states are used for security checking. The SCM signal can be generated using any of the various modulation techniques proposed in CV-QKD research to encode variables with weak coherent states, e.g., discrete modulation^36^ or Gaussian modulation^37^. Displacement in amplitude can be added via the method proposed in^38^.

### Monitoring accuracy in amplified and unamplified links

In a manner similar to that for QKD post-processing, the QA monitoring accuracy is also influenced by the finite size effect^39^. We can derive the monitoring accuracy based on the length of the data. For an unamplified link, the accuracy can be written as:

1 $$T\sim \left[ {(\hat t - Z_{\frac{{{\it{\epsilon }}PE}}{2}}\sqrt {\frac{{\sigma ^2}}{{mV_A}}} )^2{\mathrm{/}}\eta ,(\hat t + Z_{\frac{{{\it{\epsilon }}PE}}{2}}\sqrt {\frac{{\sigma ^2}}{{mV_A}}} )^2{\mathrm{/}}\eta } \right]$$

2 $$\xi \sim \left[ {\hat \xi - Z_{\frac{{{\it{\epsilon }}PE}}{2}}\frac{{\sigma ^2\sqrt 2 }}{{T\eta \sqrt m }},\hat \xi + Z_{\frac{{{\it{\epsilon }}PE}}{2}}\frac{{\sigma ^2\sqrt 2 }}{{T\eta \sqrt m }}} \right]$$

where *V*_*A*_ is the modulation variance of the quantum signal, *m* is the monitoring block length, $Z_{\frac{{{\it{\epsilon }}PE}}{2}}$ is the confidence level and *σ*^2^ is the unknown noise variance and is given by $\sigma ^2 = 1 + \eta T\,\xi + V_{ele}$. The noise variance is normalized to the pre-calibrated system shot noise units (snu).

Normally, in quantum key distribution, fibre amplifiers cannot be used to extend the transmission distance because the excess noise destroys the quantum information stored in the quantum states and introduces security loopholes^40^. Only a single pre-amplifier can be used to compensate for the efficiency loss of the detector. The modelling of the noise introduced into quantum states by amplifiers has been studied extensively; see refs. ^41,42^. Here, we consider only a quantum-noise-limited phase-insensitive amplifier (PIA)^43,44^, which adds a minimal 2(*g*^2^ − 1) vacuum noise units for a given amplitude gain *g*, and a classical amplifier (EDFA)^45^ that adds $2n_{sp}(g^2 - 1)$ unit of shot noise, where *n*_*sp*_ is the population inversion coefficient.

To analyse the overall performance, we assume an amplified channel that consists of several segments of 50 km each, where the amplifier compensates for the fibre loss and the total gain is unity. The data encoded in the quantum states after *n* fibre spans at the receiver can be modelled as:

3 $$y_n^\prime = (g\sqrt T )^n\sqrt \eta x + z^\prime$$

where $g\sqrt T = 1$ and z’ is a noise term that follows a normal distribution with variance $\sigma _n^{\prime 2}$, which can be written as:

4 $$\sigma _n^{\prime 2} = 2n_{sp}\left( {g^2 - 1} \right) + g^2T\eta \left( {\xi + \sigma _{n - 1}^{\prime 2}} \right) + V_{ele}$$

**Fig. 2:**
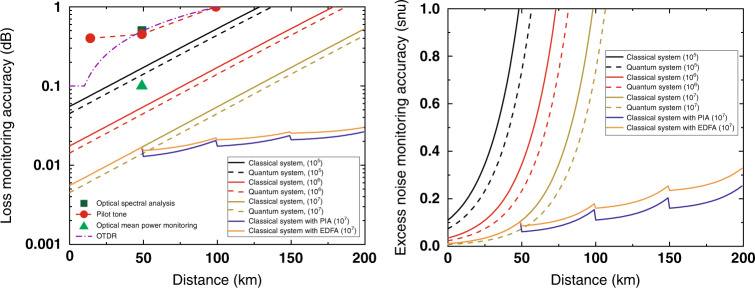
Monitoring uncertainty at different distance. Monitoring uncertainty for channel loss (**a**) and quantum excess noise (**b**). For the classical system, the electronic noise is 0.4 shot noise, and the excess channel noise is 0.1 shot noise. For the quantum system, the electronic noise is 0.015 shot noise, and the excess channel noise is 0.01 shot noise. The PIA is a quantum-limited phase-insensitive amplifier. The inversion factor of the EDFA is set to 1.5. The detector efficiency is set to 0.4. The accuracy results for the classical monitoring system are as follows: ±0.1 dB for optical mean power monitoring (OMPM)^2^, ±0.5 dB for optical spectral analysis (OSA)^3^, 0.4 dB at 50km for a pilot tone^3^, and moderate sensitivity at a short distance (0.1 dB) but poor sensitivity at a long distance (0.05 0dB/dB)^4,9^ for OTDR. The noise is normalized to units of system shot noise (snu)

The simulation results for both amplified and unamplified links are shown in Fig. 2, where we calculate the monitoring accuracy for the two parameters of interest as functions of distance for monitoring block lengths of 10^5^, 10^6^, and 10^7^ with different system parameters. We consider two different receiver system conditions: (i) a typical classical system receiver whose system parameters are taken from a classical communication system, which has a high bandwidth (>10 GHz) and is relatively low in cost, and (ii) a quantum system receiver whose parameters are taken from the CV-QKD system (>10 MHz) in^35^, which is relatively expensive.

For an unamplified link, we can see that the monitoring data block length and loss are the major factors that influence the QA monitoring performance and that the QA system is comparable to the classical system. In addition, the excess noise monitoring performance drops exponentially with distance, with an uncertainty exceeding one snu at longer distances, while the loss monitoring performance remains better than that of the best classical monitoring system (±0.1 dB) at 100 km.

For an amplified link, in terms of loss monitoring, the monitoring accuracy is better than ±0.02 dB after three stages of amplification (200 km) and is far superior to that of the classical monitoring methods, e.g., OTDR, whose accuracy is approximately ±0.05 dB/dB^4^. Regarding the excess noise monitoring performance, the improvement is even more obvious. We predict a monitoring accuracy of 0.2 shot noise units at 200 km. As a result, we find that the additional excess noise does not cause the QA monitoring performance to degrade. This is because, although the amplification adds extra noise, a noisy version of the quantum signal does not cause the accuracy to degrade as severely as the loss. This is a suprising result that shows excellent potential for the application of the QA approach in amplified optical links.

### Proof-of-principle experiment

**Fig. 3:**
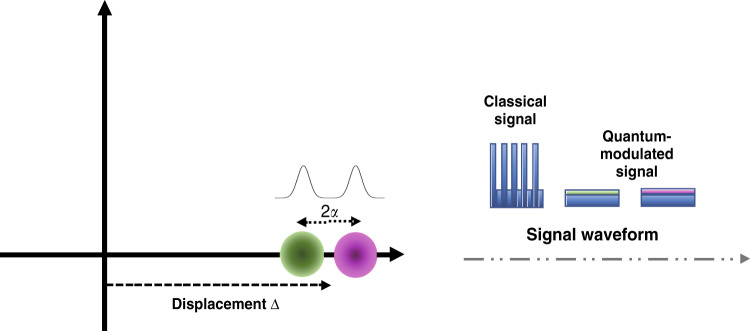
Phase-space illustration of displaced two-state modulation and the signal waveform. 2*ɑ* is the difference between the two displaced coherent states alpha and beta. This is equivalent to two-state modulation with a modulation variance of 2*ɑ*^2^. The variance of the one coherent state, which is represented by a Gaussian distribution, is one shot noise unit. The slots for the SCM and CCM are deterministic, and the shown signal pattern is repeated over time. The first time slot is for the CCM, where on-off keying is employed to encode 10 bits. The next two slots are for the SCM, where two quantum states are transmitted. The first quantum state, alpha, has a slightly larger amplitude than the second state, beta

The first demonstration of the monitoring performance using the quantum-modulated signal is for a channel with a 10 dB loss. The monitoring uncertainty and how it changes during an emulated fibre tapping attack are tested. For simplicity, in this proof-of-principle demonstration experiment, we employ the displaced two-state modulation scheme, which is equivalent to two-state modulation^46,47^, as proven in ref. ^31^. This modulation can be effectively generated with a single amplitude modulator. In addition, we send quantum signals periodically with pre-shared knowledge of which time slots are designated for the SCM. The equivalent modulation variance is calculated as V_A_ = 2α^2^, where α is the difference between the two states, as illustrated in Fig. 3. The post-processing method is the same as the Gaussian modulation scheme. In the experiment, the modulation variance is set to 20 snu, i.e., $\alpha = \sqrt {10}$.

**Fig. 4:**
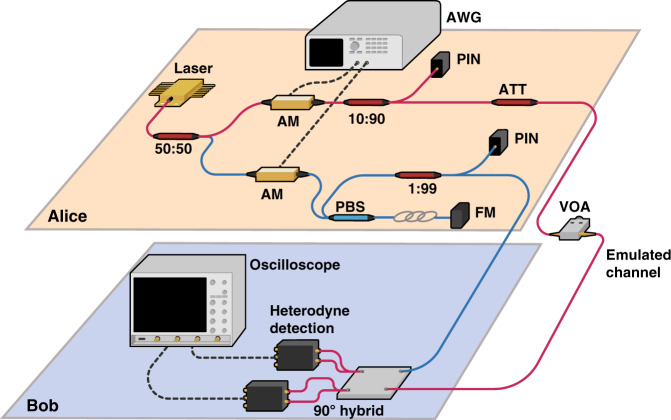
Proof-of-principle experimental set-up. AM: amplitude modulator, VOA: variable optical attenuator, ATT: attenuator, PBS: polarization beam splitter, FM: Faraday mirror. A 1550 nm CW laser is used to generate both the QA signal and LO light pulses with a slot repetition rate of 25 MHz. On the signal path, an amplitude modulator is used to generate the two-state modulated SCM signal as well as the on-off keying data in the slot for classical communication (CCM). The light is then attenuated to reduce the difference between the two states for quantum level modulation. A 10/90 splitter is used to split off 10% of both the QA signal light and the LO light for power monitoring by a photodiode. A variable optical attenuator is employed for channel emulation. For the LO path, the CW laser is pulsed at the same repetition rate. A Faraday mirror and a delay line are added to stabilize the LO polarization and match the LO pulse and QA signal pulse at the receiver. The heterodyne detector measures both the X and P quadratures of the QA signal. The oscilloscope records the measured quadrature components and also the LO and signal power fluctuations

Hence, as shown in Fig. 3, the quantum signal has two very close levels, which we refer to as states alpha and beta, transmitted in succession. We send the QA signal and the classical signal in different time slots with pulse widths of 10 ns and slot durations of 40 ns. Hence, the repetition rate of the quantum signal is 25 MHz, and the data rate of the classical signal is 1 Gb/s. The set-up for realizing the monitoring scheme is illustrated in Fig. 4. A detailed introduction to the set-up and the calibration process can be found in the methods section.

Notably, as a result of small variations in the physical environment, the fibre channel characteristics change slightly over time. To evaluate the monitoring accuracy, we first characterize the factors that influence the received signal, which include the channel characteristics, the input signal fluctuations at the transmitter, the LO power fluctuations at the receiver, and the detector imbalance.

**Fig. 5:**
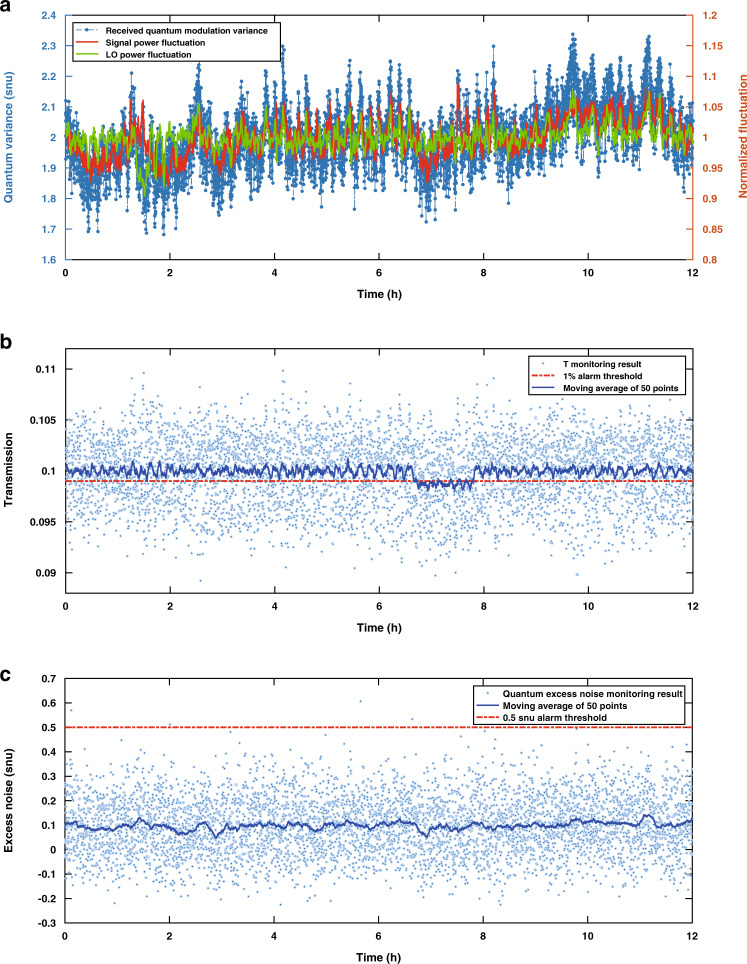
Experimental results of the system performance and 1% fibre tapping attack detection. **a** Received signal, input power fluctuations and LO power fluctuations over 12h. Each point is calculated as an average of 10^5^ pulses. For each pulse, we acquire one point and plot all measured quantum modulation variance averages. The data are collected every 8s for 12h. The LO power and input power fluctuations are also plotted with a block size of 10^5^. The power is normalized to their mean value. **b** Channel transmission monitoring results and 1% fibre tapping attack demonstration. The blue dots show the actual monitoring results when the block size is 10^5^, while the solid line shows the moving average of 50 rounds. The red dashed line is the 1% alarm threshold. A fibre tapping attack is demonstrated between hours 6 and 8. **c** Excess quantum noise monitoring results. The mean system excess noise is 0.1 snu. The 0.5 snu alarm threshold is also depicted

First, to remove fluctuations in the output current for heterodyne detection caused by the LO and signal fluctuations, we continuously monitor the input power and the LO power by using two photodetectors to measure 10% of the LO light and signal light. In addition, the quantum efficiencies of the two photodiodes inside one balanced detector will be slightly different in practice. We balance them by slightly misaligning the detector with the higher η to reduce its efficiency to match the lower one. We also test the responses of the two balanced detectors, which should also be close to ensure stable heterodyne detection. We then consider that the remaining fluctuations are fluctuations caused by channel characteristics, which cannot be reduced unless averaged over a longer time, and fluctuations caused by monitoring estimation uncertainty, which can only be reduced by increasing the block length. In the experiment, we run the system at a repetition rate of 25 MHz. At this rate, we can potentially check the link security 250 times per second with a data block length of 10^5^ and a pulse width of 10 ns. The performance in the initial experiment is limited by the connection between the scope and the PC, taking 8 s to transfer 1 second’s worth of data. To overcome this, we run the system overnight for 12 h with T equal to 0.1, i.e., with the loss of the VOA equal to 10 dB. The results are plotted in Fig. 5a–c.

### Robust performance and 1% fibre tapping attack detection

The results have been normalized to snu. In Fig. 5a, we plot the received quantum signal modulation variance, the input signal, and also the LO fluctuations. The received average difference between the two quantum states is 2$\sqrt {snu}$, i.e., a modulation variance of 2 snu after channel loss. The fluctuations of the signal and LO pulse power have been normalized and also plotted. The real-time loss and excess noise monitoring results obtained by employing the calculations introduced previously and removing influential factors are illustrated in Fig. 5b, c.

In Fig. 5b, we present the monitoring results over 12 h; the mean transmission is 0.10, while the mean excess quantum noise is 0.1 snu. For clarity, we also illustrate the smoothed 50-point moving average for both parameters. From the previous section, for a block length of 10^5^, we learn that the statistical error of 6.5 standard deviations for the excess quantum noise is 1 snu, which is even larger than the measured value (±0.78 snu). Hence, we can infer that the fluctuations in the measured excess quantum noise are mainly caused by the statistical estimation. However, for channel transmission monitoring, the measured standard deviation is ±0.1 dB, while the statistical accuracy is only approximately ±0.4% (0.02 dB) when the channel loss is 10 dB. Hence, we can estimate that the transmission deviation of the channel over 8 s is approximately Δ*T* = 0.08 dB when *T* = 10 dB. This indicates that the monitoring uncertainty comprises only a small part of the measurement fluctuations.

We also demonstrate a sophisticated fibre tapping attack by adding a 1/99 splitter into the emulated channel for 4000 s. As seen from Fig. 5b, between hour 6 and hour 8, the average link transmission drops significantly for 4000 s, indicating a possible fiber tapping attack. The alarm thresholds for 1% fibre tapping and a 0.5 snu increase in excess quantum noise are also shown. The fibre tapping alarm threshold is triggered when the moving average of the transmission crosses the threshold for a certain period. We cannot detect the attack from the excess quantum noise because it is calculated in reference to Alice’s side, where the influence of the channel transmission is cancelled out. However, we can still detect the attack from the channel transmission monitoring results. Notably, if an eavesdropper were to attempt to resend a classical signal of zero, we would still see a drop in the channel transmission. In addition, if a sophisticated eavesdropper were to measure the quantum signal and resend a replica, i.e., perform an intercept-resend attack, we would witness an increase of two shot noise units in the excess noise monitoring results. Thus, one can identify and characterize an attack by detecting different statistical characteristics of our monitoring result distributions.

A good quantum monitoring protocol should enable Alice and Bob to communicate their entire message when there is no eavesdropper, i.e., avoid false alarms, and to lose only a small amount of information when there is an eavesdropper, i.e., achieve quick response. This could be accomplished by exploring various methods of statistical change point detection^48^, e.g., Bayesian change point detection^49^, a supervised learning algorithm^50^, or CUSUM^51^. In the Supplementary Information, we analyse one method using the moving average. In the illustrated experimental results, we can thus be more than 99.96% sure that the detected event is caused by an eavesdropper, with the QA system taking less than 0.2 s to detect the attack. This result is impressive, as the QA system detects a small change of 0.1% with a very fast response when the channel loss changes from 10% to 9.9% by processing the quantum modulation variance, while the average number of received photons is more than 30,000 per pulse.

## Discussion

We have presented a new application of a quantum communication system, i.e., a quantum alarm (QA) system. It is able to detect all classes of known physical layer attacks that target classical communications links, including eavesdropping and jamming attacks, and can achieve a much faster security monitoring response than classical methods with very high accuracy, better than 0.02 dB at 200 km for loss monitoring, which is much higher than the accuracy of classical methods (±0.1 dB at 50 km)^1^, and better than 0.2 snu for excess quantum noise monitoring. In this work, a QA system has been implemented using a technique based on CV quantum communications.

A QA system solely monitors a quantum signal for suspicious changes. As a result, in comparison to a CV-QKD system (which is very sensitive to excess channel noise and receiver system noise^52^ and has relatively high requirements in terms of the system properties), a QA system is potentially more compatible with current optical infrastructure, e.g., the use of optical amplifiers and DWDM. In addition, a QA system can be easily introduced for high-data-rate communication links of up to hundreds of kilometres in length. Security is achieved on the basis of identifying statistical changes in the received quantum states.

We have demonstrated the first working system using this technique to protect a Gbps classical communication link with a channel loss of up to 10 dB and stable performance over up to 12 h. We have performed a classical fibre tapping attack of 1%, which can be precisely detected by the QA system. In practice, by adjusting the monitoring block length, the ratio between the numbers of slots for security checking and classical communication, which determines the accuracy of attack identification and the time taken to identify an attack, can be adjusted for different application requirements. Compared to QKD, in which a very long block length is required for channel estimation, a relatively short block length of 10^5^ can enable fast reaction to attacks.

QKD was proposed in response to the vulnerabilities of conventional cryptography in the face of future technology, i.e., quantum computers. However, practical challenges^53^, e.g., the key rate at a long distance, the system complexity and the incompatibility with current optical networks, still restrict the use of QKD methods in current large-scale optical communication networks. In addition, current eavesdroppers still rely on classical attack methods, which unavoidably introduce noise and a considerable level of additional loss. The QA technique provides another option for protecting information secrecy by ensuring physical layer security. Future technical advancements may enable Eve to deploy a highly sensitive detection system that will allow her to circumvent the eavesdropping detection threshold of a QA system, thus making the current proposal ineffective. However, future QA systems will also be able to utilize other technological advancements, such as ultra-low-noise lasers, homodyne detectors and highly parallel data processing for parameter estimation from very large data samples, to improve the detection sensitivity.

In practice, a QA system can be used in cooperation with other encryption methods to minimize the information obtained by an eavesdropper before the triggering threshold is reached. Various statistical change point detection methods can also be explored for attack detection in QA systems. In addition, the merits of compact classical transceivers, e.g., small-form-factor pluggable transceivers, can also be exploited for QA commericialization.

## Methodology

### Parameter estimation

Specifically, Bob first compares the quadrature he measures with Alice’s and then estimates the covariance matrix of the shared states. This is accomplished by means of the following linear model, in which Alice’s quadrature values $x_{i = 1 \ldots m}$ and Bob’s received quadrature values $y_{i = 1 \ldots m}$ are linked through^39^:

5 $$y_i = tx_i + z$$

where $t = \sqrt {T\eta }$ and *z* follows a centered normal distribution with unknown variance $\sigma ^2 = 1 + \eta T\xi + V_{ele}$. Note that for simplicity, $x_{i = 1 \ldots m}$ and $y_{i = 1 \ldots m}$ represent all of the quadrature values that Alice and Bob share, including both the X and P quadratures. In addition, *η* and *V*_*ele*_ are the efficiency and electronic noise variance, respectively, of the receiver. The channel transmission *T* and the excess noise *ξ* can be expressed as shown in the following equations:

6 $$T = \frac{{x_iy_i^2}}{{\eta Var(x_i)^2}}$$

7 $$\xi = \frac{{Var(y_i)}}{{\eta \hat T}} - Var\left( {x_i} \right) - \frac{{N_0}}{{\eta \hat T}} - \frac{{V_{ele}}}{{\eta \hat T}}$$

Hence, based on these equations, these two parameters can be estimated and regularly monitored by Bob by performing real-time post-processing of the measurement outcomes associated with the quantum signals.

### Finite size effect

In the system, the correlated data obtained by Alice and Bob, $(x_i,y_i)_i = 1...m$, are linked through:

8 $$y_i = tx_i + z$$

where ${\mathrm{t}} = \sqrt {{\mathrm{T}}\eta }$ and *z* follows a centred normal distribution with unknown variance $\sigma ^2 = 1 + \eta T\,\xi + V_{ele}$. Unbiased estimators $\hat t$ and $\hat \sigma ^2$ are known for the normal linear model:

9 $$\hat t = \frac{{\mathop {\sum}\nolimits_{i = 1}^m {x_iy_i} }}{{\mathop {\sum}\nolimits_{i = 1}^m {x_i^2} }}$$

10 $$\hat \sigma ^2 = \frac{1}{m}\mathop {\sum}\limits_{i = 1}^m {(y_i - \hat tx_i)^2}$$

where *m* is the number of data encoded. The maximum-likelihood estimator $\hat t$ follows a normal distribution, and $\hat \sigma$ has a chi-squared distribution:

11 $$\hat t\sim N\left( {t,\frac{\sigma }{{\mathop {\sum}\nolimits_{i = 1}^m {x_i^2} }}} \right)$$

12 $$\frac{{m\hat \sigma ^2}}{{\sigma ^2}}\sim \chi ^2(m - 1)$$

The accuracy of the estimation can be analysed simply by calculating the confidence intervals of *t* and *σ*:

13 $$t\sim \left(\hat t - Z_{\frac{{{\it{\epsilon }}PE}}{2}}\sqrt {\frac{{\sigma ^2}}{{mV_A}}} ,\hat t + Z_{{\it{\epsilon }}PE/2}\sqrt {\frac{{\sigma ^2}}{{mV_A}}} \right)$$

14 $$\sigma ^2\sim \left(\hat \sigma ^2 - Z_{\frac{{{\it{\epsilon }}PE}}{2}}\frac{{\sigma ^2\sqrt 2 }}{{\sqrt m }},\hat \sigma ^2 + Z_{\frac{{{\it{\epsilon }}PE}}{2}}\frac{{\sigma ^2\sqrt 2 }}{{\sqrt m }}\right)$$

where *V*_*A*_ is the modulation variance of the quantum signal *Var*(*x*_*i*_) and $Z_{\frac{{{\it{\epsilon }}PE}}{2}}$ is the confidence level. We can thus write the estimated upper and lower bounds on the two monitoring parameters as follows:

15 $$T\sim \left[ {\left(\hat t - Z_{\frac{{{\it{\epsilon }}PE}}{2}}\sqrt {\frac{{\sigma ^2}}{{mV_A}}} \right)^2{\mathrm{/}}\eta ,\left(\hat t + Z_{\frac{{{\it{\epsilon }}PE}}{2}}\sqrt {\frac{{\sigma ^2}}{{mV_A}}} \right)^2{\mathrm{/}}\eta } \right]$$

16 $$\xi \sim \left[ {\hat \xi - Z_{\frac{{{\it{\epsilon }}PE}}{2}}\frac{{\sigma ^2\sqrt 2 }}{{T\eta \sqrt m }},\hat \xi + Z_{\frac{{{\it{\epsilon }}PE}}{2}}\frac{{\sigma ^2\sqrt 2 }}{{T\eta \sqrt m }}} \right]$$

### Experimental set-up

As illustrated in Fig. 4, on Alice’s side, a 1550 nm continuous-wave (CW) laser source is split into two paths: the signal path and the LO path. For the signal path, each light pulse is 10 ns long, with a repetition rate of 25 MHz. In addition, for the classical signal, we use binary intensity modulation. Hence, the quantum and classical signals are generated using one amplitude modulator. The data patterns are illustrated in Fig. 5b, where the classical signal and the quantum-modulated signal, states alpha and beta, are transmitted sequentially. It should be noted that the classical signal has a higher bandwidth and that 10 bits are transmitted in the time taken for one of the quantum pulses, with a data rate of 1 Gb/s. A 10/90 splitter directs 90% of the input light to a photodiode that continuously measures and monitors the input power fluctuations. The remaining optical signal is attenuated to 1 μW before being connected to a variable attenuator, which is used to emulate the channel. The LO light is also modulated by an amplitude modulator to generate a pulsed signal with the same repetition rate and pulse width as the signal. Because the LO light and signal pulses originate from the same laser, the signal and LO pulses are coherent and cause minimal detection-induced noise. In this initial experiment, for simplicity, the LO signal is sent along a separate path. To avoid time delay mismatch, the LO path is engineered to be the same length as the signal path through the insertion of a variable fibre delay. All of the components in the set-up are polarization-maintaining components to ensure stable detection of the quantum signal.

On the receiver side, a heterodyne receiver is used that consists of one 90-degree optical hybrid detector and two balanced detectors. The balanced detectors are homodyne detectors intended for classical coherent communication, with an input power limit of 5 mW. The heterodyne detector measures both the X and P quadratures of the received signal. As illustrated in Fig. 4a, since the two-state quantum signal is displaced in the phase space, the modulation can be seen as unidimensional, and the quantum information is stored in the amplitude of the quantum states. Although the relative phase of the quantum state and the LO reference will vary along the channel, we can thus measure the encoded variables by simply taking the magnitude of the vector sum of the two measured quadratures at the receiver. In addition, the LO power is approximately 300 μW, which results in a photon number of 10^8^ photons per pulse. The level of the displaced quantum signal is reduced to approximately 1 μW (−30 dBm) before the emulated channel.

## Supplementary information

Supplementary information for secure optical communication using a quantum alarm

## Data Availability

Additional data related to this publication is available at 10.17863/CAM.56391.
