# Supplementary material for: Secure optical communication using a quantum alarm
Source: Light Sci Appl. 2020 Oct 2;9:170. doi: 10.1038/s41377-020-00409-1 (PMC7532184; doi:10.1038/s41377-020-00409-1)
Supplement: Supplementary file 1 — Supplementary information for secure optical communication using a quantum alarm [file 41377_2020_409_MOESM1_ESM.pdf]

Supplementary information: Secure optical communication using a quantum alarm

Yupeng. Gong[1]*, Rupesh. Kumar[2], Adrian. Wonfor[1]*, Shengjun. Ren[1], Richard. V. Penty[1]* and Ian. H. White[1,3]

[1]*Centre for Advanced Photonics and Electronics, 9 JJ Thomson Ave, University of Cambridge, Cambridge, CB3 0FA*

[2]*Quantum Communications Hub, Information Centre, Department of Physics, University of York, York, YO10 5DD*

[3]*University of Bath, Claverton Down, Bath, BA2 7AY*

**Correspondence: Yupeng Gong, Adrian Wonfor, Richard. V. Penty*

yg311@cam.ac.uk, aw300@cam.ac.uk, rvp11@cam.ac.uk

1. False alarm probability

The ideal condition for QA monitoring occurs when Alice and Bob are communicating through a lossless and noiseless channel, so that any increase in excess noise or loss is due to eavesdropper. However, in practice, given the channel intrinsic noise and loss fluctuations, a good quantum monitoring protocol should enable Alice and Bob to communicate the entire message when there is no eavesdropper, i.e. avoid false alarm, and losing only a small amount of information when there is eavesdropper, i.e. quick response. We first analysis a potential fiber tapping attack. For a stable fibre channel, the average loss can be seen as stable over a long period of time. However, the instantaneous loss fluctuates with time due to fibre characteristics. Hence, we model the instantaneous channel loss monitoring result as a Gaussian distribution with mean $\mu = \mu_0$ and unknown standard deviation σ_0 that consists of channel fluctuation and estimation uncertainty.

When there is a fibre tapping attack, the mean is shifted to μ_1 ($\mu_1 < \mu_0$) with the same fluctuation σ_0. Bob must therefore distinguish between the two Gaussian distribution or the following two hypotheses:

$$\text{H0: (Fibre tapping attack=No)} => \mu \geq \mu_0$$

$$\text{H1: (Fibre tapping attack=Yes)} => \mu \leq \mu_0$$

We can thus construct the estimator as:

$$v = \frac{\bar{t} - \mu_0}{S/\sqrt{n}} \sim t(n-1)$$

where n is number of security checking rounds, S is the measured fluctuation and $\bar{t}$ is the average value of the monitoring result. In our demonstration experiment, the measured fluctuation S is 3%, 1%, 0.3%, (with respect to the average value), for the block length of 105, 106, 107. The hypothesis H0 is rejected if and only if:

$$v < -t_\alpha(n-1)$$

where $-t_\alpha(n-1)$ is the αth quantile of the student's distribution t of n-1 degree freedom.

Hence α is also the probability that an alarm is triggered due to channel fluctuation, i.e. false alarm probability $P_{False\ alarm}$ which should be made sufficiently low, e.g. (10^{-4}). The alarm threshold should be set for the sake of classical communication. For example, the least amount an eavesdropper needs to decode the information or achieve a certain BER. Some physical encryption methods for classical communication can be used in corporation with QA to set the secure threshold. In our demonstration experiment, we set the alarm threshold for a fibre tapping attack as 1% which is usually enough for an eavesdropper to have a reliable BER for an OOK system. Hence if we set the alarm threshold, we can then calculate the false alarm probability as:

$$P_{False\ alarm} = \int_{-\infty}^{\frac{\mu_1-\mu_0}{S/\sqrt{n}}} \frac{\Gamma(\frac{\nu+1}{2})}{\Gamma(\frac{\nu}{2})} \frac{1}{\sqrt{\nu\pi}} \frac{1}{(1+\frac{x^2}{\nu})^{\frac{\nu+1}{2}}} dx$$

where ν is the degree of freedom and $\Gamma(\mathrm{x})$ is the Gamma function.

The same analysis can be performed for the quantum excess noise and we can calculate the false alarm probability as illustrated in figure S1(b). Thanks to the quantum level sensitivity, for an intercept-resend attack [1] that only introduces two units of shot noise can be easily detected with the system. It will be even easier for QA system to detect a classical jamming attack that influence the classical communication.

As illustrated in the figure S1(a), the false alarm probability drops dramatically with the number of

rounds increases. For instance, in our demonstration, we can be more than 99.96% sure that the channel is not safe after we detected the moving average result of 50 rounds of security checking (block length=10^5) crosses the 1% threshold and the alarm should be raised. In addition, if we require the false alarm probability should be less than 99.99%, for a system with security checking repetition rate of 100MHz, with the SCM/CCM ratio of 0.1, an attack cannot last longer than 0.62 seconds for 10^5, while the same took 1 seconds and 3 seconds for 10^6, and 10^7 block length. For quantum excess noise monitoring, block length of 10^5 also has the best performance that can react within 0.05 seconds for an intercept-resend attack.

To sum up, we can distinguish an attack based on the different statistical characteristics of our monitoring result distribution. This problem is also known as the statistical change point detection [2]. Many advanced algorithms have been proposed, e.g. Bayesian change point detection [3], the supervised learning algorithm [4], CUSUM [5], for faster and more accurate detection. These methods can also be explored for the QA system which is, however, beyond the scope of this paper.

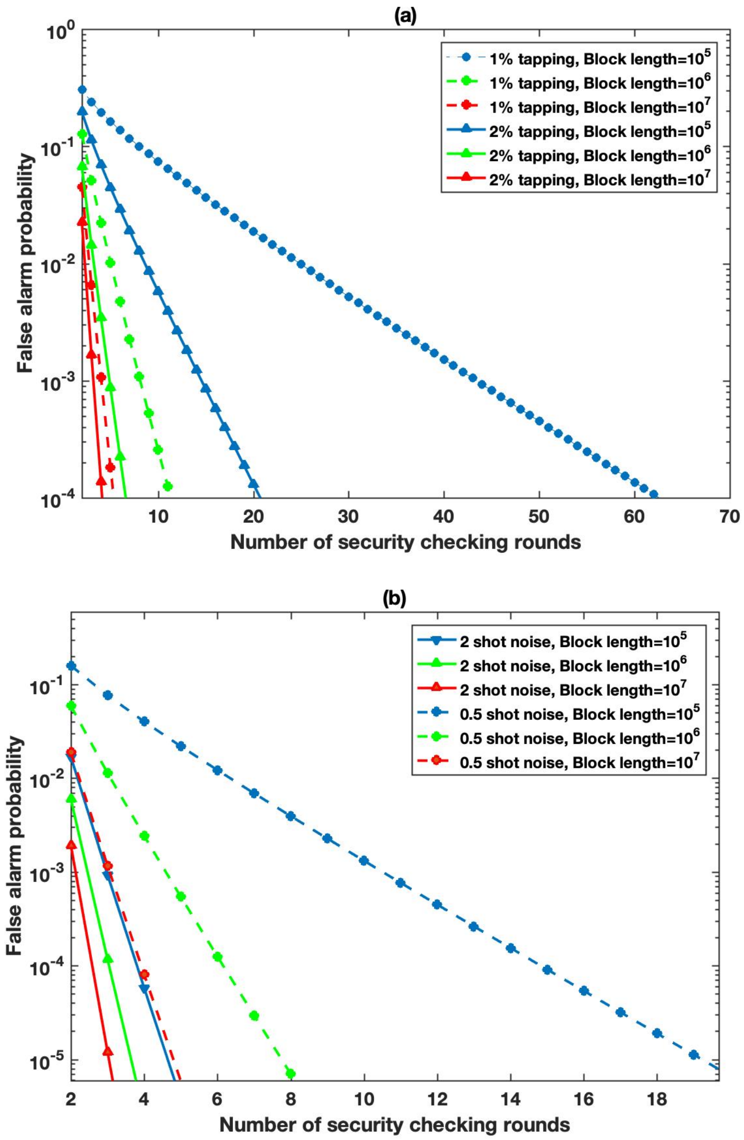

Figure S1: (a) False alarm probability when after n rounds security checking of fibre tapping attack of 1% and 2%. The red, blue, and black lines are for the block length of 10^5,10^6, 10^7. For instance, we are 99.99% sure to detect a fiber tapping attack of 1% after 62 rounds of the security check. The calculation is performed for a channel of 10dB loss and channel fluctuations are measured in previous experiment. **(b) False alarm probability for quantum excess noise monitoring.** The solid line is for an intercept-resend attack which is a non-Gaussian attack that introduces 2 units of shot noise and the dashed line is for an ideal partial intercept-resend attack that taps 25% and introduce 0.5 unit of shot noise.

2. Quantization noise on quantum detection caused by displacement

In contrast to normal quantum detection system, our quantum signal is displaced in amplitude. In order to detect the small quantum level modulation, the relatively large amplitude (displacement) will add extra measurement noise ε_m due to the finite dynamic range $[-x_m, x_m]$ of detector [6, 7], given by:

$$\varepsilon_m = \frac{1}{N_0\sqrt{2\pi V_B}}\int_{-\infty}^{-x_m}(x + x_m)^2 e^{-\frac{(x-\alpha\prime)^2}{2V_B}}\,dx + \frac{1}{N_0\sqrt{2\pi V_B}}\int_{-\infty}^{-x_m}(x - x_m)^2 e^{-\frac{(x-\alpha\prime)^2}{2V_B}}\,dx$$

If we choose a classical detector with sufficient bandwidth and dynamic range, the extra detection noise is negligible compared with the detector electronic noise, which is in the order of 10^{-9} snu. In our principle demonstration system, the detector we employed is designed for classical coherent communication which has a input power limit of 5mw.

However, the quantization noise remains a problem. The quantization noise is due to the finite resolution of the Analog to Digital Converter (ADC) which is given by [8]

$$\varepsilon_q = \frac{1}{N_0}\left[0.5 \times \frac{x_m-(-x_m)}{2^M}\right]$$

where M is the number of bits of the ADC. For instance, in our system, we have to increase the scale of the digital oscilloscope whose bit number is 10, if we increase the amplitude of a pulse. However, this decreases the precision of the measurement. The relationship between the quantization noise and received QA signal strength is plotted in figure S2.

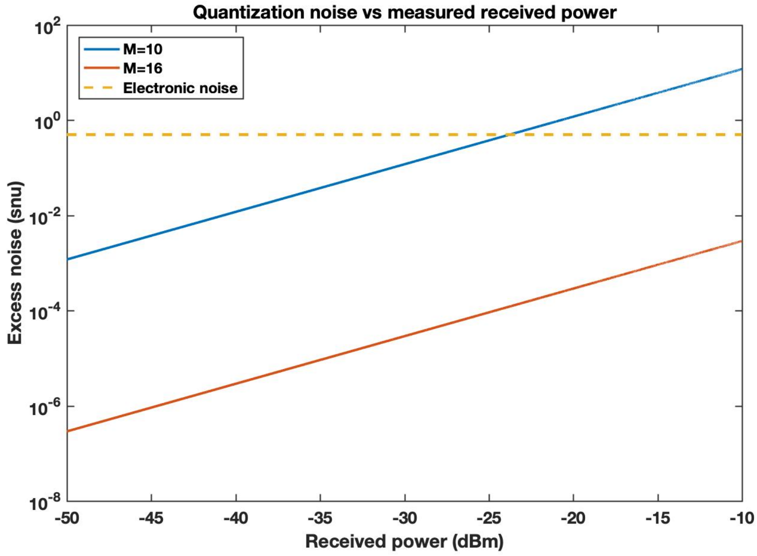

Figure S2: Theoretical quantization noise vs received monitoring signal power. The simulation is calculated for a detection system of efficiency 0.4, detector electronic noise of 0.5 shot noise, signal wavelength at 1550 nm, signal pulse duty cycle of 0.25, and ADC card bit number M of 10 or 16.

As it can be seen from figure S2, when the received quantum signal strength exceeds -20 dBm, the quantization noise exceeds the detector electronic noise (0.5 snu). This power is in the classical communication level. Hence in practice, we can displace the quantum signal to the average zero level of the classical signal. We can also employ an ADC card with a sufficient number of bits, so that the quantization noise is negligible. We also measure the quantization noise for our detector system. This is achieved by repeating the shot noise/electronic measurement process with different oscilloscope scale. We repeat the process for scale values of 5 mV, 10 mV, 20 mV, 50 mV, 150 mV and 200 mV with the same input conditions. As we have measured the precise results of the shot noise and electronic noise in the previous measurement, the increase in the measured noise for the scale is considered as from the quantization noise. By subtracting the baseline noise, we can see the influence of the quantization on the shot noise and electronic measurement and infer the maximum input power we can have while having an accurate measurement.

The results are shown in figure S3. As can be seen from the figure, the quantization noise remains at a negligible level when the resolution is less than 50 mV. This leads to a total range of 400 mV, which is enough for our detector system detecting an input optical signal level of less than -30 dBm. However, if we want to increase the displacement further, the quantization noise will exceed the electronic noise and cannot be neglected.

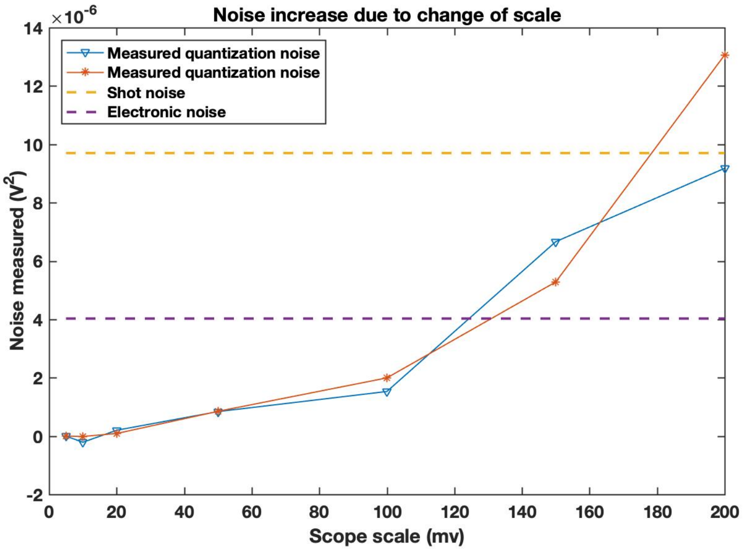

Figure S3: Experimental measured quantization noise vs Oscilloscope scale. The oscilloscope ADC card has a bit number of 10.

Reference

1. Lodewyck, J., et al. *Experimental implementation of non-gaussian attacks on a continuous-variable quantum key distribution system*. in *2007 Quantum Electronics and Laser Science Conference*. 2007.
2. Aminikhanghahi, S. and D.J. Cook, *A Survey of Methods for Time Series Change Point Detection.* Knowledge and information systems, 2017. **51**(2): p. 339-367.
3. Prescott Adams, R. and D.J.C. MacKay, *Bayesian Online Changepoint Detection.* 2007: p. arXiv:0710.3742.
4. Li, F., G.C. Runger, and E. Tuv, *Supervised learning for change-point detection.* International Journal of Production Research, 2006. **44**(14): p. 2853-2868.
5. Severo, M. and J. Gama. *Change Detection with Kalman Filter and CUSUM*. 2006. Berlin, Heidelberg: Springer Berlin Heidelberg.
6. Chi, Y.-M., et al., *A balanced homodyne detector for high-rate Gaussian-modulated coherent-state quantum key distribution.* New Journal of Physics, 2011. **13**(1): p. 013003.
7. Qin, H., R. Kumar, and R. Alléaume, *Quantum hacking: Saturation attack on practical continuous-variable quantum key distribution.* Physical Review A, 2016. **94**(1): p. 012325.
8. Qi, B., *Simultaneous classical communication and quantum key distribution using continuous variables.* Physical Review A, 2016. **94**(4): p. 042340.
